# Traumatic Hip Dislocation with Associated Femoral Head Fracture

**DOI:** 10.1155/2015/865786

**Published:** 2015-03-19

**Authors:** H. Dortaj, A. Emamifar

**Affiliations:** Golestan Hospital, Tehran 1668644611, Iran

## Abstract

Dislocation of the hip is a critical injury that results from high-energy trauma. This paper describes a case of posterior dislocation of the right hip in a 35-year-old woman with associated ipsilateral femoral head fracture. Initial treatment included reduction of the right hip through posterior approach and fixation of the femoral head fracture with three absorbable screws. After 15-month follow-up, a full range of motion has been achieved and there are no signs of avascular necrosis, hip instability, or limping. The authors describe their method of surgery.

## 1. Introduction

Traumatic hip dislocation is an infrequent and important injury that results from high-energy trauma. Associated injuries are common and they will influence the final outcome. These include fractures of the femoral head, neck or shaft fractures, acetabular fractures, pelvic fractures, knee injuries, ankle and foot injuries, or a combination of these. The vast majority of hip dislocations occur from motor vehicle accidents [1, 2].

Hip dislocation is categorized into anterior and posterior dislocation. Posterior dislocation is much more common than anterior dislocation (90% postdislocation). Two original classification schemes have been described for posterior dislocation [3]. Epstein's type 5 dislocation includes femoral head fracture that has been subdivided by Pipkin into four types as follows [4, 5]:

 type I: fracture below the fovea not involving weight-bearing surface of the head,
 type II: fracture above the fovea involving weight-bearing surface of the head,
 type III: type I or II fracture with associated femoral neck fracture,
 type IV: type I or II fracture with associated acetabulum fracture.

A hip dislocation or fracture dislocation is an orthopedic emergency. Time of presentation and more importantly reduction of the hip dislocation are essential in treating this injury and minimizing long-term complications, such as avascular necrosis of the femoral head and late osteoarthritis of the hip. The authors present a case of traumatic posterior hip dislocation with associated femoral head fracture (Pipkin type 2).

## 2. Case Report

**Figure 1:**
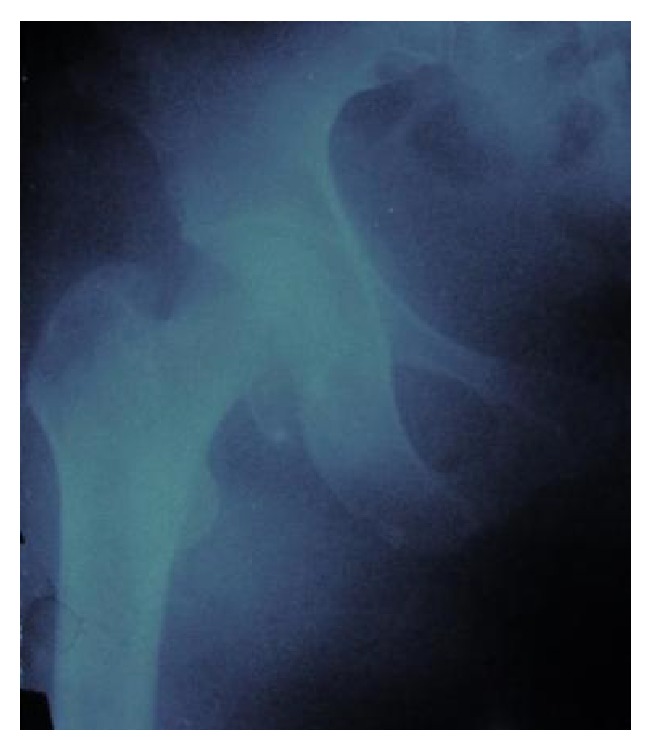
Radiograph revealed the fracture of the femoral head.

**Figure 2:**
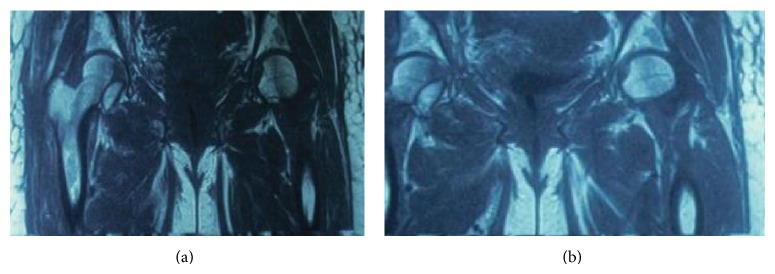
MRI showed fragments of the femoral head.

**Figure 3:**
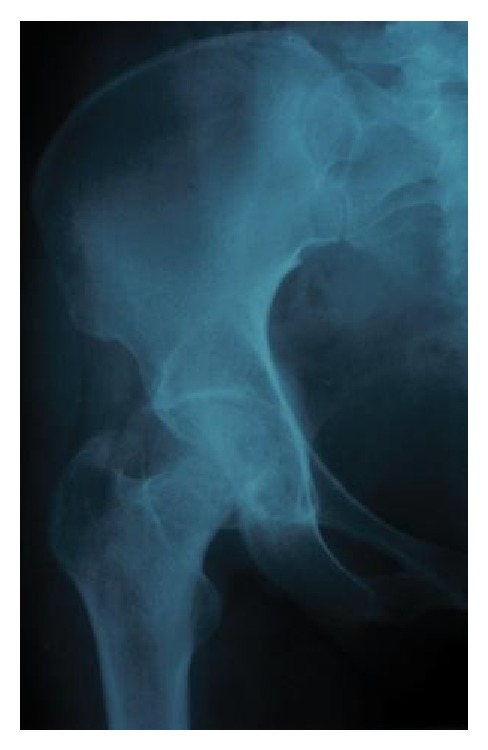
Radiograph of hip.

**Figure 4:**
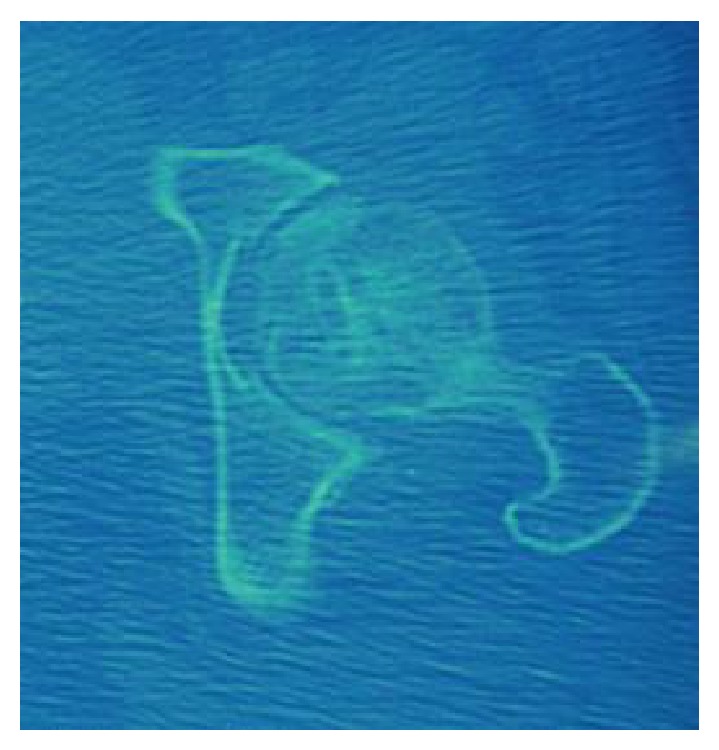
CT scan of hip.

**Figure 5:**
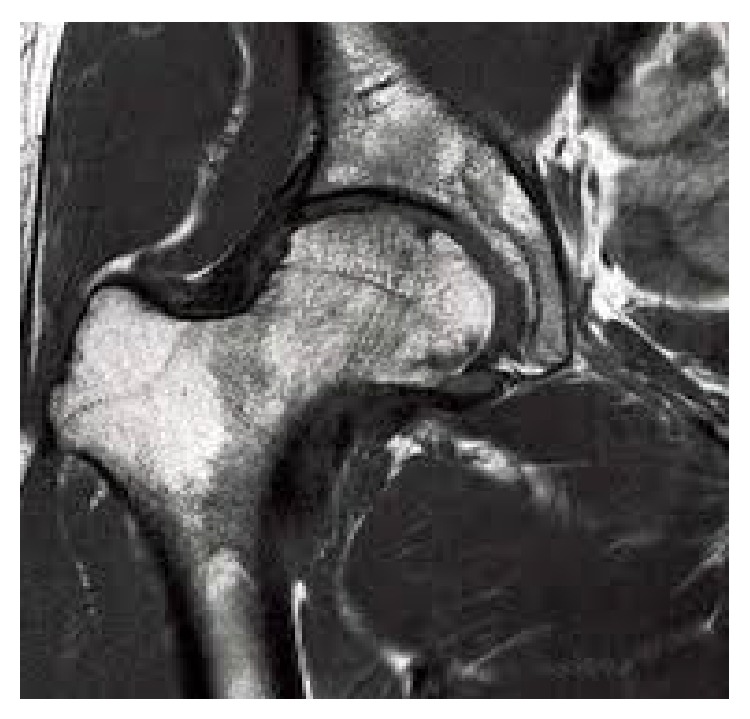
MRI of hip.

In February 2012, a 35-year-old female suffered from a high-velocity accident. She had been unable to weight bear since the injury. The patient was transferred to the nearest hospital by EMS. On admission, the injury was closed and there were no accompanying injuries. Popliteal and ankle pulses were palpable on the right foot and neurosensory examination was normal. Her blood pressure and pulse rate were within normal limits. No other injuries or skin lesions except bruising were noted on physical examination. Direct radiographs revealed posterior dislocation of right hip. This kind of injury often occurs when the knee hits the dashboard in a collision. This force drives the thigh backwards, which drives the ball head of the femur out of the hip socket. Closed reduction was performed under general anesthesia and she was discharged from the hospital. Four days later, the patient presented to our hospital with a complaint of limitation of motion in the right hip. Her general condition was stable. Further radiographs (Figure 1) and magnetic resonance imaging of the hip (Figures 2(a) and 2(b)) were performed and showed a large fragment of the femoral head fracture that was unreduced with incongruity of hip joint. At operation, the hip was exposed through a posterior approach. The short external rotators (gemelli and obturator internus) were preserved during the approach. Hip capsule rupture was clearly seen. At operation, the femoral head was taken out from acetabulum with minimal rotation and maneuver through the ruptured capsule of hip joint. The femoral head split to three parts, two large fragments and a small one. Under direct observation, we reduced fragments and fixed them preliminarily with small pins. The pins were over-drilled with a cannulated drill bit. Then with countersink, the entry site was deepened a few millimeters near the screw head size. Consecutively, the fracture was fixed with three large absorbable screws. Good spherical head and fixation were achieved. Finally the operated femoral head was reduced. Postoperatively, the patient was mobilized non-weight-bearing on crutches for eight weeks. At monthly follow-up, good union of fracture parts and spherical femoral head with no signs of avascular necrosis have been seen. At the latest follow-up (15 months after injury), radiographs (Figure 3), computed tomography (Figure 4), and MRI (Figure 5) of the hip showed no evidence of avascular necrosis (AVN) of the femoral head. She had a full range of motion with no hip instability, pain, or limping.

## 3. Discussion

Hip dislocation is a rare injury, requiring a massive force to occur. The incidence of long-term complications such as avascular necrosis (AVN) following dislocation of the hip varies from 6% to more than 40%. The rate of AVN may be affected by the time the femoral head remains dislocated. Occasionally, congruent reduction may not be achieved because of small cartilaginous or osseous fragments that remain in the joint space. In a traditional manner, immediate closed reduction is the treatment of choice based on Thompson and Epstein's classification. In cases of unsuccessful closed reduction, with fragments trapped in the joint after reduction, or associated fractures of the femur, open reduction is required [6, 7]. Dreinhofer et al. published an 8-year follow-up of 50 cases of simple dislocation of hip. They showed that the most important factors in the long-term prognosis appear to be the direction of the dislocation and the overall severity of injuries [5]. Upadhyay et al. in a cohort study of 74 cases of simple traumatic dislocation of hip reported that 24% of dislocated hips went on to develop osteoarthritis after 14.65 years. They presented that the maximal incidence of osteoarthritis was among miners aged between 31 and 40. Upadhyay and Moulton in another follow-up study of 91 cases suffering from posterior dislocation of hip demonstrated that various methods of initial pretreatment, such as traction, and non-weight-bearing for various periods influenced short-term outcome, characterized by earlier restoration of hip joint function, better pain relief, and higher rates of prevention of avascular necrosis. However, on the long term the grade of initial trauma was the most important factor in producing osteoarthritis. There is general agreement that the more severe the initial injury, the greater the chance of osteoarthritis [8, 9]. Abdulaziz et al. reported in a retrospective study of 58 cases of traumatic hip dislocation that road traffic accidents are the most common cause of traumatic hip dislocation. Rockwood's preferred treatment is performing open reduction anteriorly and fixation with Herbert screws placed underneath the articular surface [10].

We chose to combine the posterior approach in order to allow access to the hip and fixation of the ipsilateral femoral fracture with three absorbable screws. Our surgical protocol of fixation of the femoral head fractures using the posterior approach was applied because the posterior hip joints structures were already damaged by trauma. This allowed us to use this route for accessing the femoral head and acetabulum without any additional surgical trauma (iatrogenic). Due to that, we observed and manipulated the femoral head more easily [11]. Delayed treatment may result in avascular necrosis and collapse of the articular surface in this type of injury. The advantage of this approach in posterior dislocation of hip is having an easier access to the femoral head fractures from the damaged posterior hip joint area without inflicting any new trauma to the hip joint. This allows for easier reduction and fixation. Furthermore, if avascular necrosis develops in future, there is no need for hardware removal.
